# Effects of Dietary High-Yield Protease *Bacillus subtilis* Strain FRE76 on Broiler Growth, Slaughter Performance, Intestinal Morphology, and Gut Microbiota

**DOI:** 10.3390/ani15081085

**Published:** 2025-04-09

**Authors:** Liping Sun, Haihong Bi, Xinyuan Hu, Xi Chen, Yating Li, Huijing Niu, Caixia Pei, Jing Zhang, Qiang Liu, Jianhui Li, Chengqiang Xia

**Affiliations:** College of Animal Science, Shanxi Agricultural University, Jinzhong 030801, China; slp268975@163.com (L.S.); 18436916828@163.com (H.B.); hxy_16@163.com (X.H.); chenxi7592@163.com (X.C.); liyating417@163.com (Y.L.); huijingniu@163.com (H.N.); caixiapeisxnd@163.com (C.P.); zhangjingdongke@163.com (J.Z.); liuqiangabc@163.com (Q.L.); jianhui19840717@163.com (J.L.)

**Keywords:** broilers, *Bacillus subtilis*, growth, slaughter performance, intestinal morphology, gut microbiota

## Abstract

This study aimed to investigate the effects of supplementing broiler feed with high-yield protease *Bacillus subtilis* strain FRE76 on growth performance, slaughter performance, apparent digestibility, intestinal morphology, and intestinal microbiota. One-day-old Arbor Acres broilers (n = 240) were randomly assigned to four groups (n = 6 replicates; 10 animals per replicate), in which their basal diet was supplemented with *B. subtilis* strain FRE76 at 0 CFU/kg (group C), 3.60 × 10^8^ CFU/kg (group L), 1.08 × 10^9^ CFU/kg (group M), and 1.80 × 10^9^ CFU/kg (group H). The experiment lasted for 42 d and comprised two phases: Days 1–21 and days 22–42. The broilers’ body weight at 21 d and the average daily gain at 1–21 d in the L and H groups increased significantly relative to those in group C (*p* < 0.05). The half-bore weight, half-bore percentage, and breast muscle percentage in group L were significantly higher (*p* < 0.05) than those in group C. The apparent digestibility of crude protein in group L and the ether extract in groups L, M, and H were significantly increased at 22–42 d compared with those in group C (*p* < 0.05). In group L, the jejunal villus height and villus height/crypt depth increased significantly relative to those in group C (*p* < 0.05). The chymotrypsin and trypsin activities tended to increase in the *B. subtilis* FRE76 groups (*p* = 0.072 and *p* = 0.056, respectively) relative to those in group C. Additionally, the abundance of *Bacteroidota*, *Proteobacteria*, *Alistipes*, *Clostridia*_vadinBB60_group, and *Parabacteroides* increased significantly in the *B. subtilis* FRE76 groups (*p* < 0.05). In conclusion, dietary *B. subtilis* FRE76 could improve broilers’ body weight, average daily gain, apparent digestibility, protease activity, intestinal morphology, and gut microbiota.

## Simple Summary

There is a worldwide attempt to reduce or ban the use of feed antibiotics to promote animal growth, and researchers are constantly investigating alternative techniques for poultry production. Probiotics, as a kind of green feed additive, have been utilized in animal and poultry farming for decades because of their beneficial effects on animal growth and health. *Bacillus subtilis* is one of the most common probiotic strains for animal production. In our previous study, a high-yielding protease *B. subtilis* strain was screened and isolated from the cecal contents of Shanxi Bian chickens. Thus, the present study aimed to explore the effects of adding different concentrations of high-yielding protease *B. subtilis* strain FRE76 to diets on intestinal microbiota, intestinal morphology, gut protease activity, blood biochemical indices, feed apparent digestibility, slaughter performance, and growth performance of broilers.

## 1. Introduction

Animal feed has commonly been supplemented with antibiotics [1]; however, the overuse of antibiotics has led to the emergence of resistant bacteria, concerns over food safety, and polluted environments [2]. Consequently, multiple countries have banned antibiotic supplementation of animal feed. Therefore, researchers have been investigating alternative products to augment broiler production, including herbal extracts, essential oils, exogenous enzymes, prebiotics, and probiotics [3]. Among these, probiotics have received widespread attention for their safety and effectiveness [4].

*Bacillus subtilis* is one of the most common bacterial species used in commercial probiotic products for poultry production [5]. Research has proven that diets supplemented with *B. subtilis* improve growth and nutrient digestibility [6], and enhance immunity and gut health in poultry under various rearing environments and infectious immune challenges [7,8]. However, many of the properties of probiotics vary according to the strain [9]. For example, the broiler growth performance and gut physiology effects of *B. subtilis*-containing diets were observed to be strain-dependent [10,11]. Hence, ongoing research on various strains of *B. subtilis* are needed to understand their mechanism of action in broilers.

In monogastric animals, the digestion of protein is mainly driven by endogenous proteases. Although the endogenous proteases synthesized are usually sufficient to optimize feed protein utilization, a considerable amount of protein (18–20%) passes through the gastrointestinal tract incompletely digested [12]. Moreover, in chicks, due to the incomplete development of their digestive system, the secretion of proteases is not well-coordinated with feed intake, making protein digestion more difficult and resulting in the wastage of dietary protein. A study showed that microbial protease supplementation could improve protein utilization and growth performance [13]. Therefore, we hypothesized that the addition of a high-yielding protease *B. subtilis* strain might present a series of positive effects on protein digestion and growth performance in broilers. In our previous study, a high-yielding protease *B. subtilis* was screened and isolated from the cecal contents of Shanxi Bian chickens and was named *Bacillus subtilis* FRE76, with a protease activity of 119.7 U/mL [14]. To date, no studies have focused on the effects of the addition of high-yielding protease *B. subtilis* on broilers. Thus, the present study aimed to explore the effect of adding different concentrations of high-yielding protease *B. subtilis* strain FRE76 to diets on intestinal microbiota, intestinal morphology, gut protease activity, blood biochemical indices, feed apparent digestibility, slaughter performance, and growth performance of broilers.

## 2. Materials and Methods

### 2.1. Animal Ethics

The Animal Ethics Committee of Shanxi Agricultural University granted approval of the experimental procedures. This study was carried out at the Qingxu County Yufeng Poultry Co., Ltd. (Shanxi, China).

### 2.2. Bacillus subtilis FRE76

*B. subtilis* FRE76 used in this experiment was isolated from the cecal contents of Shanxi Bian chickens. The *B. subtilis* FRE76 product for diet supplementation was prepared by activation, culture, centrifugation, and freeze-drying. The obtained product contained viable bacteria at 3.6 × 10^9^ CFU/g.

### 2.3. Experimental Design, Diets, and Management

One-day-old Arbor Acres broilers (n = 240) were randomly assigned to four groups (n = 6 replicates; 10 animals per replicate). The broilers were provided with basal diets containing *B. subtilis* FRE76 at 0 CFU/kg (group C), 3.60 × 10^8^ CFU/kg (group L), 1.08 × 10^9^ CFU/kg (group M), and 1.80 × 10^9^ CFU/kg (group H). The basal diets were formulated based on the nutrient requirements of Arbor Acres Plus broilers [15], and their nutritional components are detailed in Table 1. Before formal feeding, the chicken coop, feed tank, and site were thoroughly cleaned and disinfected. The feeding period lasted for 42 days, separated into two phases: days 1–21 and days 22–42. During feeding, the ambient temperature was maintained at 33 °C for days 1–3, followed by a gradual decrease (0.5 °C/d) to a constant 24 °C. The broilers were exposed to light for 24 h.

### 2.4. Sample Collection

The broilers’ body weight (BW) and feed intake were noted every week to calculate the average daily gain (ADG), average daily feed intake (ADFI), and feed conversion ratio (FCR). At 42 days, six broilers per group were randomly selected for euthanasia slaughter according to the average broiler weight. The production performance index was calculated according to the terminology and measurement method of poultry production performance [16].

At days 21 and 42, blood samples (5–10 mL sampled from the brachial vein) were collected from six randomly selected broilers per group. Centrifugation (3000 r/min, 10 min) was used to separate the serum, which was stored at −80 °C until use. The six broilers were then euthanized. Tissue samples of the middle intestinal segment of the jejunum and ileum (about 1.5 cm) were collected, rinsed with cold normal saline buffer, immersed in 4% paraformaldehyde solution for fixation, and used to determine their intestinal morphology. Pancreas tissue and jejunum chyme were sampled, and placed at −80 °C for subsequent determination of trypsin and chymotrypsin activities. About 1 g of cecal contents were collected into cryotubes (about 1.5 mL), snap-frozen using liquid nitrogen, and placed at −80 °C for later determination of the cecal microbiota.

### 2.5. Apparent Digestibility Measurements

During days 19 to 21 and 40 to 42, approximately 200 g of excreta were collected from each replicate and stored at −20 °C. After thawing, the excreta samples were oven-dried at 65 °C for 72 h. Then, the samples were ground to powder and passed through a 1 mm screen. Samples of diets and dried excreta were analyzed for acid-insoluble ash and apparent digestibility of organic matter (OM), dry matter (DM), crude fiber (CF), ether extract (EE), and crude protein (CP). The following equation was used to calculate the apparent total tract nutrient digestibility:

Utilization rate of a nutrient (%) = 100 − (nutrient content in excreta × acid insoluble ash content in diet)/(nutrient content in diet × acid insoluble ash content in excreta) × 100%(1)

### 2.6. Serum Biochemical Parameters Measurements

An automatic biochemical analyzer (Mindray, BS-240Vet auto-analyzer, Shenzhen, China) was used to measure the serum biochemical parameters: Uric acid (UA), urease (UREA), triglyceride (TG), aspartate aminotransferase (AST), alanine aminotransferase (ALT), low-density lipoprotein cholesterol (LDL-C), high-density lipoprotein cholesterol (HDL-C), total cholesterol (TC), albumin (ALB), and total protein (TP).

### 2.7. Intestinal Morphology Determination

The intestinal tissue was embedded, sliced, hematoxylin and eosin (HE) stained, and sealed. Imaging was performed under a P250 FLASH panoramic section scanner (Danjier, Jinan, China), and eight fields of view were taken per section. The Slide Viewer 2.6.0.166179 software (3D Histech, Budapest, Hungary) was used to measure the crypt depth (CD) and villus height (VH), and their ratio was calculated (V/C).

### 2.8. Protease Activity Measurements

The activities of trypsin and chymotrypsin were measured using a protease activity test kit (Solarbio, Beijing, China) according to the manufacturer’s protocol. Trypsin activity was defined as the absorption value at 253 nm at 37 °C per gram of tissue, which increased by 0.0005 per minute in a 1 mL system. Chymotrypsin activity was defined as the hydrolysis of 1 μmol N-Benzoyl-L-tyrosine ethyl ester per gram sample per minute at 25 °C, with one unit of enzyme activity.

### 2.9. Cecal Microbiota Analysis

Microbiota DNA was extracted from cecal samples utilizing a MagPure Soil DNA LQ Kit (Magen Biotech, Guangzhou, China) according to the supplier’s guidelines. Universal primers 515F (5′-GTGCCAGCMGCCGCGG-3′) and 907R (5′-CCGTCAATTCMTTTRAGTTT-3′) were employed to amplify the 16S rRNA genes. A Qubit dsDNA Assay Kit (Thermo Fisher Scientific, Waltham, MA, USA) was used to construct the library, which was sequenced employing an Illumina NovaSeq 6000 instrument (Illumina Inc., San Diego, CA, USA), with data processing being conducted by OE Biotech Co., Ltd. (Shanghai, China). The alpha and beta diversity analyses were performed using QIIME1 [17]. Using a linear discriminant analysis (LDA) score > 2.0, differences in bacterial taxa were identified using linear discriminant analysis effect size (LEfSe).

### 2.10. Statistical Considerations

Office Excel 2016 (Microsoft Corp., Redmond, WA, USA) was used to analyze the original data. GraphPad Prism 8.0.2 software (GraphPad Inc., La Jolla, CA, USA) was used for single-factor analysis of variance (ANOVA) and to construct the plots. Multiple comparisons were made using Tukey’s method. The analysis results were expressed as the “mean ± standard deviation”, and *p* values of <0.05 indicated significant differences.

## 3. Results

### 3.1. Growth Parameters

The details of dietary *B. subtilis* FRE76’s effects on broiler growth are provided in Table 2. Compared with group C, the BW at 21 d in groups L and H was significantly increased (*p* < 0.05), and the BW at 42 d in group L was also increased significantly (*p* < 0.05). The ADG from 1 to 21 d in groups L and H was significantly higher than in group C (*p* < 0.05). In addition, the ADG from 22 to 42 d and from 1 to 42 d in group L was augmented significantly relative to that in group C (*p* < 0.05). Among all groups in all time periods, the ADFI, FCR, and mortality rate were not significantly different.

### 3.2. Slaughter Performance

The slaughter performance results are displayed in Table 3. The half-bore weight, half-bore percentage, and breast muscle percentage in group L were significantly higher than those in group C (*p* < 0.05). However, no significant differences were observed for carcass weight, dressing percentage, full bore weight, full bore percentage, leg muscle percentage, and abdominal fat percentage among the groups (*p* > 0.05).

### 3.3. Apparent Digestibility

Supplementation of *B. subtilis* FRE76 significantly augmented the apparent total tract digestibility of EE at 21 d and 42 d (*p* < 0.05) (Table 4). Compared with that in group C, the apparent digestibility of CP in group L at 42 d was significantly higher (*p* < 0.05). The apparent digestibility of DM, OM, and CF did not differ significantly among the groups at 21 d and 42 d (*p* > 0.05).

### 3.4. Serum Biochemical Parameters

Effects of dietary supplementation with *B. subtilis* FRE76 on the serum biochemical indices of the broilers are shown in Table 5. Compared with that in group C, group L showed a significant decrease in the serum UREA content at 21 days (*p* < 0.05). Furthermore, group L showed higher serum TP, TC, and LDL-C levels compared with those in group C at 42 d (*p* < 0.05). There were no significant differences in the ALB, TG, HDL-C, ALT, AST, and UA levels of broilers among the groups at 21 d and 42 d (*p* > 0.05) (Table 5).

### 3.5. Intestinal Morphology

**Figure 1:**
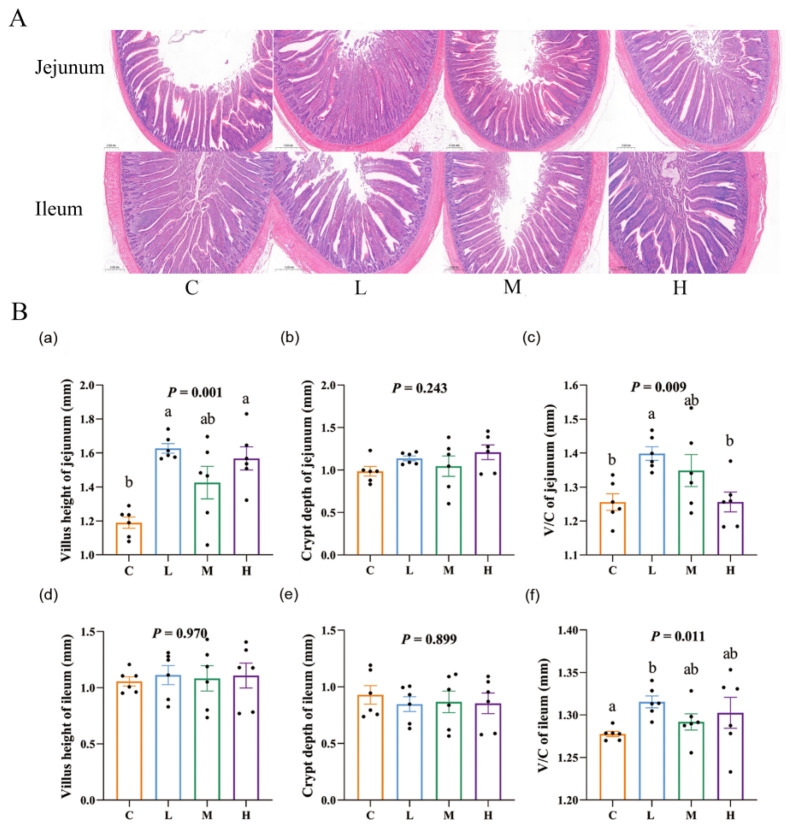
Effects of dietary supplementation of *B. subtilis* FRE76 on the intestinal morphology of broilers (n = 6). (**A**) Hematoxylin and eosin staining showing the morphological structure of the jejunum and ileum. (**B**) Villus height, crypt depth, and their ratio (V/C) in the jejunum (**a**–**c**) and ileum (**d**–**f**). Lowercase a and b mark significant differences between treatments (*p* < 0.05). Abbreviations: C, L, M, and H basal diets supplemented with 0, 3.60 × 10^8^ CFU/kg, 1.08 × 10^9^ CFU/kg, and 1.80 × 10^9^ CFU/kg *B. subtilis* FRE76, respectively. Each black dots represents an individual animal.

Figure 1 shows the morphology of the broilers’ ileum and jejunum. Compared with those in group C, the VH and V/C of the jejunum in group L were significantly increased (*p* < 0.05). Furthermore, group H showed a significant increase in the jejunal VH compared with that in group C (*p* < 0.05). Additionally, broilers in group L exhibited a higher ileal V/C than group C (*p* < 0.05).

### 3.6. Protease Activity

**Figure 2:**
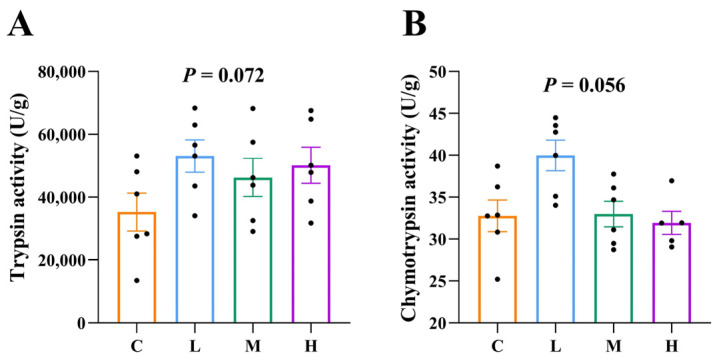
Effects of dietary supplementation of *Bacillus subtilis* FRE76 on the protease activities of broilers. (**A**) Trypsin activity; (**B**) chymotrypsin activity. Abbreviations: C, L, M, and H: basal diets supplemented with 0, 3.60 × 10^8^ CFU/kg, 1.08 × 10^9^ CFU/kg, and 1.80 × 10^9^ CFU/kg *B. subtilis* FRE76, respectively. Each black dots represents an individual animal.

The effects of *B. subtilis* FRE76 on the protease activities of broilers are shown in Figure 2. The activities of trypsin and chymotrypsin of the *B. subtilis* FRE76 supplementation groups showed an increased trend (*p* = 0.072 and *p* = 0.056, respectively).

### 3.7. Cecal Microbiota

**Figure 3:**
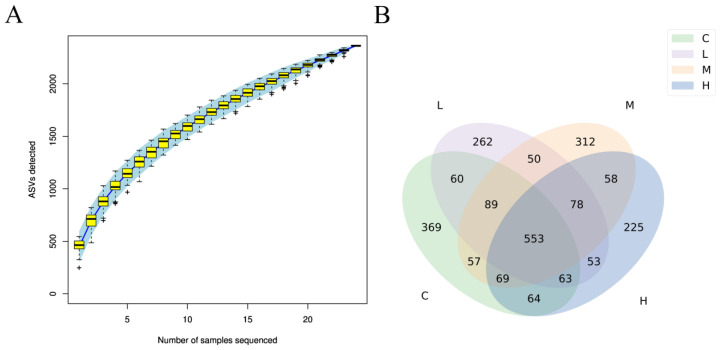
Cumulative box plot of species (**A**) and Venn diagram of the cecal microbiota (**B**). Abbreviations: C, L, M, and H: basal diets supplemented with 0, 3.60 × 10^8^ CFU/kg, 1.08 × 10^9^ CFU/kg, and 1.80 × 10^9^ CFU/kg *B. subtilis* FRE76, respectively.

**Figure 4:**
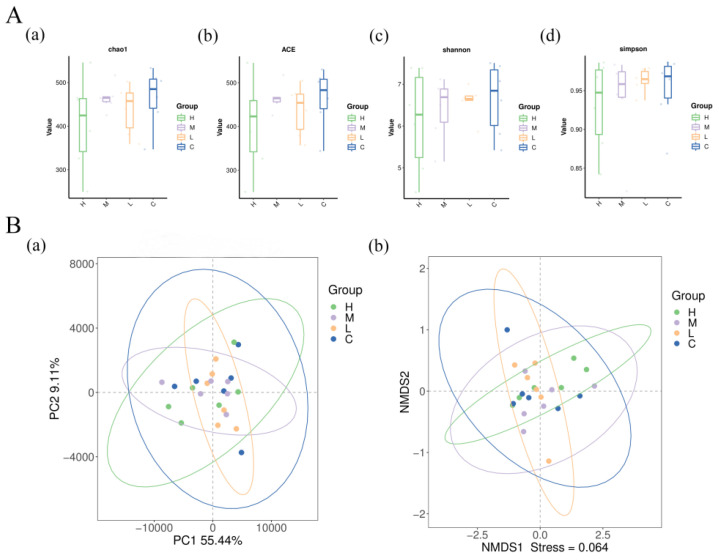
Effects of adding *B. subtilis* FRE76 to the diet on cecal microbiota. (**A**) Alpha diversity of the (**a**) Chao 1, (**b**) ACE, (**c**) Shannon, and (**d**) Simpson index; (**B**) Beta diversity analysis using principal coordinate analysis (PcoA) (**a**) and non-metric multidimensional scaling (NMDS) (**b**). Abbreviations: C, L, M, and H: basal diets supplemented with 0, 3.60 × 10^8^ CFU/kg, 1.08 × 10^9^ CFU/kg, and 1.80 × 10^9^ CFU/kg *B. subtilis* FRE76, respectively.

The species accumulation box plot tended to be flat as the number of sequenced samples reached 20 (Figure 3A), indicating that operational taxonomic unit sequences were sufficient to predict the species richness of the sample. The Venn diagram (Figure 3B) shows that groups C, L, M, and H contained 369, 262, 312, and 225 unique sequences, respectively, and there were 553 common sequences among the four treatment groups. The alpha diversity indexes (Chao1, ACE, Shannon, and Simpson) and the beta diversity (principal coordinate analysis and non-metric multidimensional scaling) are shown in Figure 4A,B. The alpha diversity showed no significant variation among the four groups. However, differences existed between group L and group H (Figure 4B).

**Figure 5:**
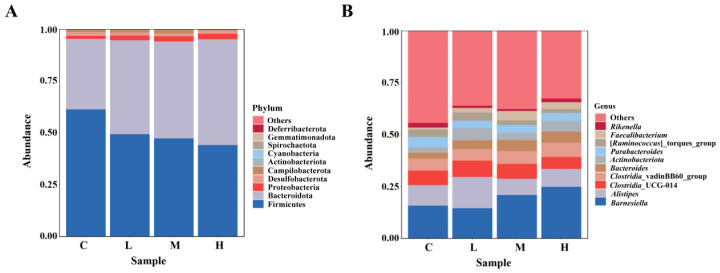
Compositions of the cecal microbiota at the phylum (**A**) and genus (**B**) levels. Abbreviations: C, L, M, and H: basal diets supplemented with 0, 3.60 × 10^8^ CFU/kg, 1.08 × 10^9^ CFU/kg, and 1.80 × 10^9^ CFU/kg *B. subtilis* FRE76, respectively.

The phylum and genus compositions of the cecal microbiota are shown in Figure 5. In the four groups, the dominant phyla were *Firmicutes*, *Bacteroidota*, *Proteobacteria*, and *Desulfobacterota*. Compared with those in group C, the levels of *Bacteroidota* and *Proteobacteria* in the *B. subtilis* FRE76 treatment groups were significantly increased (*p* < 0.05). (Table 6). At the genus level, the top 10 genera were *Barnesiella*, *Alistipes*, *Clostridia*_UCG-014, *Clostridia*_vadinBB60_group, *Bacteroides*, *Actinobacteriota*, *Parabacteroides*, [*Ruminococcus*]_torques_group, *Faecalibacterium*, and *Rikenella*. The levels of *Alistipes*, *Parabacteroides*, and *Clostridia*_vadinBB60_group were significantly increased in group L compared with those in group C (*p* < 0.05). The *Rikenella* levels in group M and the *Clostridia*_vadinBB60_group levels in group H were significantly higher relative to those in group C (*p* < 0.05) (Table 6).

**Figure 6:**
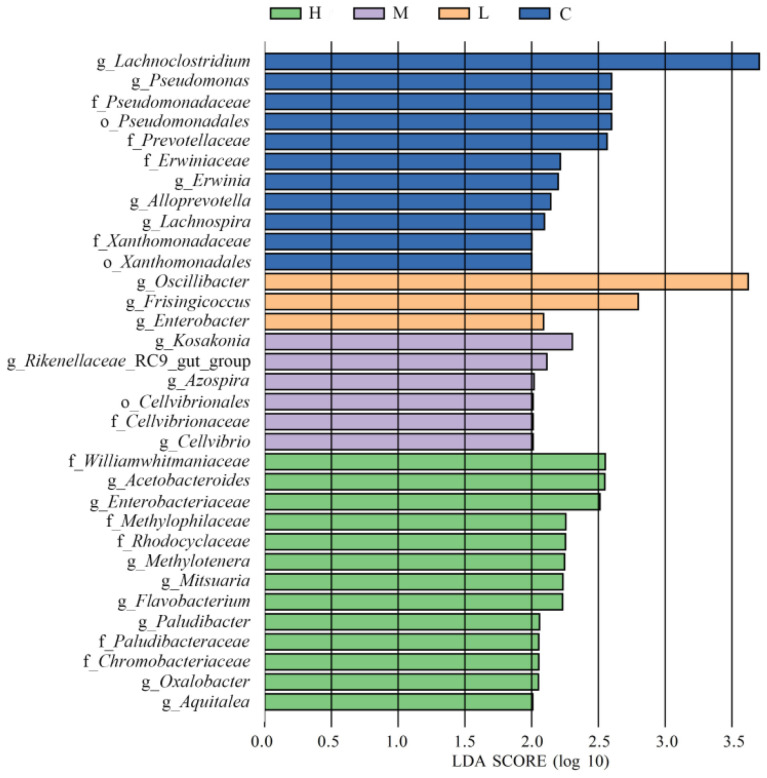
Histogram showing the linear discriminant analysis (LDA) scores for taxonomic biomarkers identified by linear discriminant analysis effect size (LEfSe). Enriched taxa are indicated by an LDA score (log 10) > 2. Abbreviations: C, L, M, and H: basal diets supplemented with 0, 3.60 × 10^8^ CFU/kg, 1.08 × 10^9^ CFU/kg, and 1.80 × 10^9^ CFU/kg *B. subtilis* FRE76, respectively.

According to the LEfSe multilevel species analysis (Figure 6), the abundances of 33 species were significantly different among the four groups. The abundances of *Oscillibacter*, *Frisingicoccus*, and *Enterobacter* were upregulated in group L. The abundances of *Kosakonia*, *Rikenellaceae*_RC9_gut_group, *Azospira*, *Cellvibrionales*, *Cellvibrionaceae*, and *Cellvibrio* were increased in group M. In group H, the abundances of *Williamwhitmaniaceae*, *Acetobacteroides*, *Enterobacteriaceae*, *Methylophilaceae*, *Rhodocyclaceae*, *Methylotenera*, *Mitsuaria*, *Flavobacterium*, *Paludibacter*, *Paludibacteraceae*, *Chromobacteriaceae*, *Oxalobacter*, and *Aquitalea* were proportionately higher.

## 4. Discussion

With the goal of developing a new probiotic that can secret beneficial metabolites, we employed the *B. subtilis* strain FRE76, which was isolated from Shanxi Bian chickens, and found to be highly efficient in protease secretion. In this study, the potential of *B. subtilis* strain FRE76 to improve growth performance, slaughter performance, apparent digestibility, serum biochemical indices, intestinal morphology, and gut microbiota of broilers was tested.

Growth performance characteristics are a series of important indicators used for evaluating the economic benefits of additives [18]. Most previous studies have demonstrated that dietary supplementation of *B. subtilis* can improve growth performance in broilers [8,19,20,21]. In this study, dietary supplementation of *B. subtilis* FRE76 had beneficial effects on the BW and ADG of broilers, indicating improved economic benefits. One possible reason for the positive effect on growth performance might be the secretion of various digestive enzymes, including protease, α-amylase, lipidase, and cellulase, by *B. subtilis* [22]. Another possibility is that *B. subtilis* could maintain the beneficial microbial population and metabolic function [23]. In addition, our results showed that *B. subtilis* increased VH, which provides a larger surface area for the effective absorption of nutrients, thereby improving nutrient absorption.

Herein, we observed that CP and EE apparent digestibility were improved significantly by *B. subtilis* FRE76. These results agree with those of Gao et al. [24], who found that dietary supplementation with *B. subtilis* increased the apparent metabolism of crude protein and crude fat. Likewise, Li et al. [25] observed that dietary *B. subtilis* significantly increased CP and EE digestibility, which was possibly related to the cumulative effect of *B. subtilis* action, including the improvement of digestive enzyme activity [26] and gut microbiota composition [27]. In this research, CP and EE apparent digestibility were most likely enhanced due to *B. subtilis*’s capacity to produce extracellular enzymes in the intestines of broilers, improving the intestinal digestion and absorption capacity of feed nutrients.

Serum biochemical indexes can reflect the health status and metabolic function of broilers. In our study, the dietary addition of *B. subtilis* FRE76 increased the levels of TP, TC, and LDL-C, but decreased UREA levels in serum. Our data matched closely with the observations of Gyawali et al. [28] and Ren et al. [29], who reported that probiotic treatments significantly increased the serum content of TP and decreased UREA. This phenomenon might be caused by *B. subtilis* supplementation inhibiting the breakdown of proteins into nitrogen and improving the absorption rate of dietary protein [30]. Interestingly, in the present study, the TC and LDL-C contents increased after *B. subtilis* FRE76 supplementation. These results differ from those of Ren et al. [29], who found that dietary supplementation with *B. subtilis* led to a decrease in serum parameters. These effects of *B. subtilis* FRE76 on blood cholesterol may be the result of enhanced cholesterol synthesis [31] or a weakening of cholesterol conversion to coprostanol by probiotics in the intestine [32].

VH, CD, and V/C are markers of broiler intestinal absorptive ability and health [33]. Increased VH and V/C could improve digestion and nutrient absorption in broiler intestines [34]. Previous studies have reported that *B*. *subtilis* supplementation improves intestinal morphology by increasing VH or V/C [35,36]. Herein, in response to *B. subtilis* addition, jejunal VH increased, and the jejunal and ileal V/C increased. Therefore, we deduced that dietary *B. subtilis* FRE76 might enhance gut health and improve nutrient absorption capacity, which could partially explain the augmented growth performance of the broilers.

Gastrointestinal digestive enzyme activity plays a crucial role in nutrient digestion, ultimately affecting broilers’ intestinal health and growth performance. Previous studies have shown that supplementation by *B. subtilis* significantly increased digestive enzyme activity, which had beneficial effects on growth performance [6,37]. In our study, supplementation with *B. subtilis* FRE76 increased intestinal trypsin and chymotrypsin activities compared with those in broilers fed the basal diet, and higher protease activity facilitates protein digestion, thereby enhancing growth performance. This is consistent with previous studies.

Various factors influence cecal microflora, which has important functions in maintaining host health and productivity, including nutrient absorption, pathogen exclusion, and immune system development [38,39]. *B. subtilis* is a gram-positive bacterium that thrives in the intestines and maintains an anaerobic environment by consuming oxygen, as well as modulating the intestinal microbiota [18,40]. Herein, the *B. subtilis* treatment groups and control group displayed no significant difference in microbiota abundance or diversity. Several studies have also reported that *B. subtilis* treatment did not affect microbiota diversity parameters [19,29,41,42]. However, Li et al. [43] reported that the addition of *B. subtilis* improved the jejunal microbiota diversity in broilers at 21 d, but not at 42 d. It is possible that the ecosystem of the intestinal microbiota is very complex, with dynamic diversity changes depending on diet and age [44]. Moreover, Zhang et al. [19] speculated that pathogen-infected chickens’ overall microbial diversity could be improved by *B. subtilis*; however, it did not significantly affect the microbial diversity of healthy birds.

*Firmicutes* and *Bacteroidota* comprised the dominant flora in all groups, accounting for more than 90% of the total flora. Our results were consistent with those of earlier studies, in which *Firmicutes* and *Bacteroidetes* were the dominant phylum in broilers, which are known to exert an important function in metabolism and energy production [6,45]. However, a study found that the dominant phylum of the broiler fecal microbiota is *Proteobacteria*, followed by *Firmicutes* and *Bacteroidetes* [46]. The difference could be attributed to the breeding environment and the age of the broilers. Moreover, an interesting phenomenon emerged in our experiments: the number of *Firmicutes* decreased significantly and *Bacteroides* levels increased significantly when broilers were fed *B. subtilis* FRE76. This is consistent with a previous study where dietary *Bacillus subtilis* BC02 supplementation reduced the abundance of *Firmicutes* but increased the abundance of *Bacteroides* [29]. Previous research has found that some members of *Firmicutes* were responsible for H_2_O_2_ production, and a reduction in *Firmicutes* led to a decrease in H_2_O_2_ levels in the cecum [47]. Higher levels of H_2_O_2_ can be toxic to both the microbes and the host because it can kill beneficial microbes and bacteria in birds [47]. Bacteroidetes showed significant responsiveness to intestinal and host environmental pressure, and their abundance correlated with short-chain fatty acid contents [48]. The changes in the number of *Bacteroides* and *Firmicutes* might be more conducive to the maintenance of the intestinal environment and the absorption of nutrients in broilers.

At the genus level, *Barnesiella*, *Alistipes*, *Clostridia*_UCG-014, *Clostridia*_vadinBB60_group, *Bacteroides*, *Ruminococcus*_torques_group, and *Parabacteroides* were the abundant bacteria in both *B. subtilis* FRE76-supplemented groups and the control group. Moreover, we found that the relative abundances of *Alistipes*, *Clostridia* vadinBB60, and *Parabacteroides* were significantly increased in the FRE76-supplemented groups. *Alistipes* (family *Rikenellaceae*) contributes to the production of succinic acid [49]. Succinic acid can be directly absorbed by broiler intestinal cells, providing cells with energy, promoting intestinal cell growth, and repairing intestinal damage [50]. Additionally, research has shown that *Alistipes* may have protective effects against some diseases such as liver fibrosis, colitis, cancer immunotherapy, and cardiovascular disease [51]. *Clostridia* vadinBB60_group is a major bacterium producing propionate [52], which has been shown to improve growth performance and feed conversion efficiency [53]. *Parabacteroides* have a wide range of cholic acid conversion functions, including the production of stone cholic acid and ursodeoxycholic acid. Stone cholic acid regulates fat absorption and metabolism, while ursodeoxycholic acid can repair intestinal wall integrity and regulate fat absorption [54]. Consequently, the improved growth performance of the broilers might have been caused, in part, by the increased levels of *Alistipes*, *Clostridia* vadinBB60, and *Parabacteroides*.

Furthermore, this study showed that group L exhibited statistically significant effects across most measured parameters, while higher probiotic concentrations failed to show significant effects. A similar finding was obtained by Abdel-Moneim et al. [30], who found that the group supplemented with 1 × 10^7^ CFU/kg *B. subtilis* in a Japanese quail bird diet had better nutrient digestibility and growth performance compared to the group supplemented with 1 × 10^9^ CFU/kg. This phenomenon may be due to the fact that concentrations of probiotics influence the constitution of the intestinal microbiota. For example, the abundance of *Alistipes* and *Parabactriodes*, which may positively affect the growth performance of the broilers, was significantly increased in group L, but not in group H. In addition, high doses of probiotics may cause host-specific adverse reactions [55], which could explain why lower doses exhibited better performance.

## 5. Conclusions

In summary, supplementing the diet of broilers with high-yield protease *B. subtilis* strain FRE76 improved their growth performance (in terms of BW and ADG), and increased the digestibility of crude protein and ether extract. The addition of *B. subtilis* FRE76 also enhanced gut health via increased jejunum villus height, an increased abundance of *Bacteroidota* and *Proteobacteria*, and a decrease in *Firmicutes*. Therefore, *B. subtilis* FRE76 appears to be a propitious option for reducing the dietary utilization of antibiotics in broilers.

## Figures and Tables

**Table 1:** Experimental diet composition and nutritional level.

Element (%)	1–21 Days	22–42 Days	Nutrient Levels ^2^ (%)	1–21 Days	22–42 Days
Corn	51.85	53.57	ME, (MJ/kg)	12.27	13.91
Soybean meal	30.00	23.89	Crude protein	26.26	22.95
Flour	8.00	8.00	Ether extract	4.20	9.27
Powdered soy	1.50	1.50	Crude fiber	1.22	2.07
Corn gluten meal	3.05	3.42	Crude ash	5.71	5.03
Duck fat	1.00	6.00	Calcium	1.01	0.83
Calcium hydrogen phosphate	1.46	0.78	Phosphorus	0.61	0.57
Fine bluestone powder	1.21	1.11	Lysine	1.00	0.80
L-Lysine sulphate	0.65	0.65	Methionine	0.65	0.55
Choline chloride	0.08	0.08	H_2_O	2.17	2.17
Premix ^1^	1.20	1.00			
Total	100.00	100.00			

^1^ Premix provided per kg of diet containing vitamin A (8000 IU); vitamin D_3_ (2000 IU); vitamin E; (20 mg); vitamin K_3_ (4.0 mg); vitamin B_1_ (2.0 mg); vitamin B_2_ (4.0 mg); vitamin B_6_ (3.0 mg); vitamin B_12_ (0.02 mg); nicotinamide (4.0 mg); folic acid (1.0 mg); biotin (0.12 mg); iron (100 mg); copper (10 mg); zinc (5.0 mg); selenium (0.2 mg); and iodine (0.2 mg). ^2^ Crude protein, ether extract, crude fiber, crude ash, calcium, phosphorus, and water comprised measured values. Metabolizable energy (ME), lysine, and methionine comprised calculated values.

**Table 2:** Effects of dietary supplementation of *Bacillus subtilis* FRE76 on growth performance of broilers.

Item ^1^	Treatment ^2^				*p*-Value
	C	L	M	H	
BW, g					
1 d	48.57 ± 0.78	47.67 ± 0.67	48.15 ± 0.71	48.36 ± 0.58	0.127
21 d	854.80 ± 7.63 ^b^	881.07 ± 9.14 ^a^	869.40 ± 16.07 ^ab^	880.50 ± 6.86 ^a^	0.005
42 d	2321.86 ± 195.79 ^b^	2613.33 ± 128.47 ^a^	2522.37 ± 130.18 ^ab^	2527.99 ± 110.39 ^ab^	0.038
ADFI, g					
1–21 d	49.01 ± 0.58	49.11 ± 0.48	49.53 ± 0.89	49.30 ± 0.85	0.705
22–42 d	116.29 ± 11.52	129.19 ± 10.72	121.17 ± 10.84	118.81 ± 8.46	0.196
1–42 d	82.67 ± 5.98	89.25 ± 5.45	85.35 ± 5.57	84.05 ± 3.94	0.195
ADG, g					
1–21 d	38.59 ± 0.34 ^b^	39.65 ± 0.36 ^a^	39.10 ± 0.76 ^ab^	39.64 ± 0.32 ^a^	0.012
22–42 d	74.50 ± 2.76 ^b^	82.55 ± 6.57 ^a^	78.49 ± 6.28 ^ab^	81.15 ± 7.17 ^ab^	0.040
1–42 d	54.23 ± 4.74 ^b^	61.06 ± 3.08 ^a^	58.91 ± 3.10 ^ab^	59.05 ± 2.56 ^ab^	0.042
FCR					
1–21 d	1.27 ± 0.03	1.23 ± 0.01	1.26 ± 0.03	1.29 ± 0.07	0.305
22–42 d	1.65 ± 0.12	1.64 ± 0.07	1.65 ± 0.17	1.65 ± 0.12	0.997
1–42 d	1.52 ± 0.07	1.48 ± 0.05	1.48 ± 0.06	1.50 ± 0.06	0.639
Mortality rate, %					
1–42 d	8.33	5.00	6.67	5.00	0.710

^1^ BW, body weight; ADFI, average daily feed intake; ADG, average daily gain; g, gram; FCR, feed conversion ratio. ^2^ C, L, M, and H: basal diets supplemented with 0, 3.60 × 10^8^ CFU/kg, 1.08 × 10^9^ CFU/kg, and 1.80 × 10^9^ CFU/kg *B. subtilis* FRE76, respectively. Data are presented as the means ± SD (n = 6), and the following table is the same. Means within a row lacking a common superscript differ (*p* < 0.05).

**Table 3:** Effects of dietary supplementation of *Bacillus subtilis* FRE76 on slaughter performance of broilers.

Item ^1^	Treatment ^2^				*p*-Value
	C	L	M	H	
CW/kg	2.25 ± 0.12	2.35 ± 0.16	2.26 ± 0.16	2.22 ± 0.14	0.147
DP/%	89.65 ± 2.24	90.20 ± 1.87	89.68 ± 4.20	89.49 ± 2.36	0.983
FBW/kg	1.79 ± 0.10	1.91 ± 0.15	1.81 ± 0.12	1.81 ± 0.07	0.082
HBW/kg	2.12 ± 0.13 ^b^	2.29 ± 0.14 ^a^	2.16 ± 0.13 ^ab^	2.14 ± 0.09 ^ab^	0.021
FBP/%	71.04 ± 1.35	72.80 ± 1.63	71.24 ± 3.38	71.67 ± 1.91	0.430
HBP/%	82.43 ± 2.61 ^b^	85.06 ± 1.32 ^a^	84.64 ± 1.67 ^ab^	84.44 ± 1.95 ^ab^	0.028
BMP/%	27.68 ± 0.35 ^b^	30.33 ± 1.22 ^a^	28.73 ± 1.22 ^ab^	28.22 ± 2.17 ^ab^	0.024
LMP/%	18.72 ± 0.58	18.02 ± 0.55	18.49 ± 0.72	18.06 ± 0.37	0.075
AFP/%	2.10 ± 0.60	2.13 ± 0.29	2.19 ± 0.56	2.35 ± 0.28	0.716

^1^ CW, carcass weight; DP, dressing percentage; FBW, full bore weight; HBW, half bore weight; FBP, full bore percentage; HBP, half bore percentage; BMP, breast muscle percentage; LMP, leg muscle percentage; AFP, abdominal fat percentage. ^2^ C, L, M, and H: basal diets supplemented with 0, 3.60 × 10^8^ CFU/kg, 1.08 × 10^9^ CFU/kg, and 1.80 × 10^9^ CFU/kg *B. subtilis* FRE76, respectively. Means within a row lacking a common superscript differ (*p* < 0.05).

**Table 4:** Effects of dietary supplementation of *Bacillus subtilis* FRE76 on the apparent digestibility of broilers.

Item ^1^	Treatment ^2^				*p*-Value
	C	L	M	H	
21 d					
DM (%)	70.45 ± 3.93	70.58 ± 2.50	70.35 ± 4.61	70.56 ± 4.20	0.996
OM (%)	74.29 ± 1.76	74.59 ± 2.27	74.27 ± 2.30	73.40 ± 3.50	0.760
CP (%)	65.47 ± 4.77	64.88 ± 2.61	66.50 ± 1.55	65.29 ± 3.66	0.923
EE (%)	81.34 ± 0.95 ^b^	83.64 ± 1.90 ^a^	84.47 ± 1.01 ^a^	83.73 ± 1.67 ^a^	0.005
CF (%)	19.45 ± 6.55	20.06 ± 6.49	20.66 ± 5.88	23.74 ± 5.63	0.556
42 d					
DM (%)	79.26 ± 1.02	80.34 ± 2.25	78.52 ± 2.05	79.05 ± 2.49	0.481
OM (%)	81.78 ± 1.89	82.35 ± 2.92	81.70 ± 1.97	82.77 ± 1.33	0.893
CP (%)	69.20 ± 2.00 ^b^	73.32 ± 2.65 ^a^	71.08 ± 2.77 ^ab^	71.71 ± 1.44 ^ab^	0.036
EE (%)	87.10 ± 2.67 ^b^	93.01 ± 2.14 ^a^	91.31 ± 0.65 ^a^	91.47 ± 1.42 ^a^	0.001
CF (%)	26.72 ± 6.85	26.76 ± 8.14	25.52 ± 6.44	27.99 ± 10.63	0.948

^1^ DM, dry matter; OM, organic matter; CP, crude protein; EE, ether extract; CF, crude fiber. ^2^ C, L, M, and H: basal diets supplemented with 0, 3.60 × 10^8^ CFU/kg, 1.08 × 10^9^ CFU/kg, and 1.80 × 10^9^ CFU/kg *B. subtilis* FRE76, respectively. Means within a row lacking a common superscript differ (*p* < 0.05).

**Table 5:** Effects of dietary supplementation of *Bacillus subtilis* FRE76 on serum biochemical indices of broilers.

Item ^1^	Treatment ^2^				*p*-Value
	C	L	M	H	
21 d					
TP (g/L)	32.32 ± 3.99	32.02 ± 1.57	34.73 ± 3.04	34.35 ± 3.20	0.515
ALB (g/L)	13.78 ± 0.98	12.32 ± 0.76	12.35 ± 1.59	14.04 ± 1.72	0.089
TG (mmol/L)	0.37 ± 0.04	0.35 ± 0.05	0.42 ± 0.14	0.38 ± 0.16	0.838
TC (mmol/L)	3.31 ± 0.29	3.42 ± 0.53	3.52 ± 0.24	3.08 ± 0.23	0.277
LDL-C (mmol/L)	0.73 ± 0.11	0.73 ± 0.13	0.67 ± 0.17	0.60 ± 0.12	0.364
HDL-C (mmol/L)	2.40 ± 0.25	2.50 ± 0.34	2.61 ± 0.20	2.24 ± 0.12	0.088
ALT (U/L)	8.20 ± 0.72	8.50 ± 1.29	6.94 ± 0.69	7.52 ± 1.10	0.102
AST (U/L)	340.75 ± 20.14	351.54 ± 62.68	340.45 ± 16.58	328.88 ± 20.36	0.794
UREA (mmol/L)	0.61 ± 0.03 ^a^	0.38 ± 0.13 ^b^	0.49 ± 0.15 ^ab^	0.57 ± 0.27 ^ab^	0.014
UA (μmol/L)	396.78 ± 66.01	413.23 ± 44.15	500.76 ± 71.46	523.43 ± 190.69	0.327
42 d					
TP (g/L)	35.04 ± 2.43 ^b^	41.84 ± 3.38 ^a^	38.38 ± 1.57 ^ab^	39.77 ± 1.68 ^ab^	0.001
ALB (g/L)	14.35 ± 1.77	14.80 ± 2.01	14.71 ± 1.50	15.15 ± 1.81	0.844
TG (mmol/L)	0.52 ± 0.04	0.61 ± 0.09	0.52 ± 0.06	0.57 ± 0.10	0.162
TC (mmol/L)	3.42 ± 0.23 ^c^	4.17 ± 0.33 ^a^	3.93 ± 0.33 ^ab^	3.66 ± 0.21 ^bc^	0.002
LDL-C (mmol/L)	0.84 ± 0.18 ^b^	1.15 ± 0.11 ^a^	1.04 ± 0.15 ^ab^	1.04 ± 0.16 ^ab^	0.018
HDL-C (mmol/L)	2.32 ± 0.16	2.51 ± 0.20	2.48 ± 0.25	2.43 ± 0.24	0.423
ALT (U/L)	5.97 ± 0.60	6.47 ± 0.66	5.91 ± 1.18	5.99 ± 1.10	0.703
AST (U/L)	565.33 ± 85.72	497.33 ± 45.99	537.53 ± 65.22	489.86 ± 73.71	0.304
UREA (mmol/L)	0.43 ± 0.15	0.45 ± 0.05	0.31 ± 0.03	0.44 ± 0.12	0.163
UA (μmol/L)	722.20 ± 98.02	627.58 ± 68.85	778.42 ± 103.13	637.98 ± 115.39	0.111

^1^ TP, total protein; ALB, albumin; TG, triglyceride; TC, total cholesterol; LDL-C, low-density lipoprotein cholesterol; HDL-C, high-density lipoprotein cholesterol; ALT, alanine aminotransferase; AST, aspartate aminotransferase; UREA, urease; UA, uric acid. ^2^ C, L, M, and H: basal diets supplemented with 0, 3.60 × 10^8^ CFU/kg, 1.08 × 10^9^ CFU/kg, and 1.80 × 10^9^ CFU/kg *B. subtilis* FRE76, respectively. Means within a row lacking a common superscript differ (*p* < 0.05).

**Table 6:** Relative species abundance at the phylum and genus levels.

Item ^1^	Treatment ^2^				*p*-Value
	C	L	M	H	
Phylum (%)					
Firmicutes	62.82 ± 11.80	52.22 ± 3.31	51.50 ± 5.71	49.12 ± 15.89	0.287
Bacteroidota	28.45 ± 10.12 ^b^	45.32 ± 7.76 ^ab^	46.73 ± 10.02 ^ab^	50.97 ± 18.88 ^a^	0.044
Proteobacteria	0.80 ± 0.43 ^b^	1.85 ± 0.54 ^ab^	1.88 ± 0.32 ^ab^	2.23 ± 1.19 ^a^	0.039
Desulfobacterota	1.46 ± 0.16	1.57 ± 0.90	1.39 ± 0.71	1.51 ± 0.53	0.975
Genus (%)					
*Barnesiella*	18.81 ± 16.40	15.34 ± 7.15	20.92 ± 2.87	27.62 ± 8.83	0.186
*Alistipes*	10.53 ± 1.58 ^b^	18.21 ± 3.43 ^a^	10.38 ± 3.80 ^b^	11.21 ± 4.68 ^ab^	0.024
*Clostridia_*UCG-014	7.29 ± 2.82	8.04 ± 4.13	7.32 ± 1.56	6.72 ± 3.04	0.935
*Clostridia_*vadinBB60_group	4.44 ± 0.97 ^b^	6.23 ± 1.12 ^a^	6.13 ± 0.72 ^ab^	7.76 ± 0.27 ^a^	0.001
*Bacteroides*	3.22 ± 0.49	4.62 ± 2.18	3.58 ± 1.01	5.36 ± 1.43	0.138
[*Ruminococcus*]*_*torques_group	5.30 ± 1.44	4.24 ± 0.40	4.43 ± 1.24	4.73 ± 0.80	0.530
*Parabacteroides*	2.11 ± 0.92 ^b^	5.51 ± 1.61 ^a^	2.17 ± 0.61 ^b^	4.65 ± 0.50 ^ab^	0.004
*Faecalibacterium*	3.46 ± 1.85	3.53 ± 0.57	3.05 ± 1.47	2.56 ± 1.60	0.769
*Rikenella*	0.73 ± 0.41 ^b^	1.36 ± 0.35 ^ab^	2.75 ± 1.15 ^a^	2.21 ± 0.73 ^ab^	0.010
*Christensenellaceae*_R-7_group	2.06 ± 1.09	1.27 ± 0.83	1.01 ± 0.38	1.05 ± 0.53	0.228

^1^ Species with relative abundance greater than 1% at phylum level and genus level. ^2^ C, L, M, and H: basal diets supplemented with 0, 3.60 × 10^8^ CFU/kg, 1.08 × 10^9^ CFU/kg, and 1.80 × 10^9^ CFU/kg *B. subtilis* FRE76, respectively. Means within a row lacking a common superscript differ (*p* < 0.05).

## Data Availability

The data that support the findings of this study are available on request from the corresponding author.
